# Cardiac and Respiratory Influences on Intracranial and Neck Venous Flow, Estimated Using Real-Time Phase-Contrast MRI

**DOI:** 10.3390/bios12080612

**Published:** 2022-08-08

**Authors:** Maria Marcella Laganà, Alice Pirastru, Francesca Ferrari, Sonia Di Tella, Marta Cazzoli, Laura Pelizzari, Ning Jin, Domenico Zacà, Noam Alperin, Giuseppe Baselli, Francesca Baglio

**Affiliations:** 1 IRCCS Fondazione Don Carlo Gnocchi ONLUS, 20148 Milan, Italy; 2 Department of Electronics, Information, and Bioengineering, Politecnico di Milano, 20133 Milan, Italy; 3 Department of Psychology, Università Cattolica del Sacro Cuore, Largo A. Gemelli, 1, 20123 Milan, Italy; 4 MR R&D Collaborations, Siemens Medical Solutions USA, Inc., Cleveland, OH 44106, USA; 5 Siemens Healthcare, 20128 Milano, Italy; 6 Department of Radiology, University of Miami Miller School of Medicine, Miami, FL 33136, USA

**Keywords:** RT-PC MRI, intracranial venous flow, neck venous flow

## Abstract

The study of brain venous drainage has gained attention due to its hypothesized link with various neurological conditions. Intracranial and neck venous flow rate may be estimated using cardiac-gated cine phase-contrast (PC)-MRI. Although previous studies showed that breathing influences the neck’s venous flow, this aspect could not be studied using the conventional segmented PC-MRI since it reconstructs a single cardiac cycle. The advent of real-time PC-MRI has overcome these limitations. Using this technique, we measured the internal jugular veins and superior sagittal sinus flow rates in a group of 16 healthy subjects (12 females, median age of 23 years). Comparing forced-breathing and free-breathing, the average flow rate decreased and the respiratory modulation increased. The flow rate decrement may be due to a vasoreactive response to deep breathing. The respiratory modulation increment is due to the thoracic pump’s greater effect during forced breathing compared to free breathing. These results showed that the breathing mode influences the average blood flow and its pulsations. Since effective drainage is fundamental for brain health, rehabilitative studies might use the current setup to investigate if respiratory exercises positively affect clinical variables and venous drainage.

## 1. Introduction

Many factors influence the intracranial and neck venous return: first of all, the heart contraction and the amount of arterial flow to the brain [1]; second, the periodic thoracic pressure changes due to respiration [2,3,4,5]; and third, the posture [3,6,7]. While the venous flow rate’s periodic oscillations due to the heart’s beating have been well described for decades [8,9], the modulations due to respiration have been only recently assessed using ultrasound [2,3] and MRI [5,10,11]. The clinical segmented cine phase contrast (PC) MRI acquires one segment of k-space lines in one heartbeat. It requires several heartbeats to form time-resolved flow images covering the entire cardiac cycle. (PC) MRI has been used for assessing the intracranial and extracranial venous flow and its relationship with arterial and cerebrospinal fluid flows [9,12,13]. Nonetheless, it does not allow the detection of beat-by-beat flow rate changes and the identification of respiratory influences. The recent advent of a real-time (RT)-PC MRI technique for flow quantification allowed evaluation of the effect of coughing, inspiration, and expiration on the venous flow [5,10,11] and beat-by-beat variability [14]. However, the published studies investigated the blood flow rate of extracranial vessels only, with the exception of work from a preliminary conference proceeding [15] that showed intracranial maps. To the best of our knowledge, only a previous ultrasound study by Zamboni et al. [2] specifically assessed the effect of respiration on intracranial venous flow. Their results showed that a deep inspiration, compared to normal breathing, increases the blood return from the brain. They noted that the internal jugular vein (IJV) flow parameters changed, but not those of the intracranial parenchymal veins and dural sinuses, the former being close to the chest and thus more influenced by the thoracic pump.

Nevertheless, since the flow velocities obtained using ultrasound are operator-dependent, our study aimed to quantify the effect of respiration on intracranial and neck venous flow using RT-PC MRI, a technique that allows quantification of the venous flow in real time.

We focus on IJVs as representative of the main extracranial venous flow pathway in the supine position. For intracranial drainage, we considered the superior sagittal sinus (SSS), this being the largest dural venous sinus and receiving venous blood from different superficial cortical veins in both the cerebral hemispheres [16,17], and given its fundamental role in cerebrospinal fluid drainage and waste removal [18]. The study of brain drainage is fundamental due to its implications in the neurological field, as venous abnormalities, cerebral blood flow, and cerebrospinal fluid flow alterations have been described in neurodegenerative [19,20,21,22,23] and neuroinflammatory diseases [22,24,25,26,27,28]. Moreover, the link between the age-related brain’s venous circulation alterations and the pathogenesis of vascular cognitive impairment and dementia is an open issue that is gaining much scientific interest [29].

In this work, we estimated the IJV and SSS flow rates using the RT-PC MRI, and we tested whether different breathing modes influenced: (1) the average flow rate, and (2) the amount of cardiac and respiratory modulations.

## 2. Materials and Methods

### 2.1. Subjects

Sixteen healthy subjects (12 females and 4 males, median age: 23 (range 19–57) years) were recruited. Inclusion criteria were the absence of neurological, neuropsychiatric, and cardiovascular disorders, as assessed via an ad hoc self-reported health questionnaire. The study was performed in accordance with the principles of the Helsinki declaration and was approved by the IRCCS Fondazione Don Gnocchi Ethical Committee. Written informed consent was signed by each participant.

### 2.2. MRI Acquisitions

The acquisitions were performed on a 3T clinical scanner (MAGNETOM Prisma, Siemens Healthcare, Erlangen, Germany) equipped with a 64-channel head–neck coil.

The scanning protocol for the IJV and SSS flow rate measurements consisted of two separate PC sequences, one with a velocity encoding (VENC) rate of 70 cm/s and the other with a VENC of 40 cm/s, respectively. Both were run using a RT-PC prototype sequence [14,30] with a segmented EPI readout, parallel acceleration factor in the temporal direction, and a 2-sided shared velocity encoding reconstruction algorithm (field of view [FOV] = 153 × 175 mm^2^, matrix size = 96 × 128, interpolated resolution = 0.7 × 0.7 mm^2^, slice thickness = 8.6 mm, time resolution of 58.5 ms, GRAPPA = 3, TR/TE = 14.6/8 ms, flip angle = 15°) [14]. For IJVs acquisition, the imaging slice was positioned at the C1 vertebra level, perpendicularly to the vessels. For the SSS image acquisition, the slice was positioned perpendicularly to the sinus, visualized in a sagittal plane. The VENC of each RT-PC sequence was selected with the preliminary acquisition of two subjects, experimentally choosing the minimum VENC that did not produce aliasing. For the IJVs, we tested the VENC = 70 cm/s following the method used in [9] with satisfactory results. For the SSS, we tested various VENCs from 20 cm/s to 50 cm/s with a 10 cm/s step, finally selecting the VENC of 40 cm/s. We expected that with this VENC, aliasing might occur for some subjects in some pixels of the vessel, where the velocity is the highest, typically during systole. However, we tried to minimize aliasing and could correct it when we processed our PC images, as explained below.

Each RT-PC sequence was acquired for 60 s, and was repeated during 3 different breathing scenarions: (1) free breathing (F), i.e., the subject was asked to follow his/her spontaneous breathing rate; (2) paced normal breathing (PN), i.e., the subject was instructed to breathe at a constant rate, as explained hereafter; (3) paced deep breathing (deep PD), i.e., similar to the previous scenario (2) but while forcing inhalation and exhalation. To maintain a constant breathing rate, in scenarios (2) and (3) we used a visual stimulus consisting of a circle with an enlarging/decreasing diameter as guidance. Both the BRs of (2) and (3) were adapted to each subject. Regarding (2), we simply imposed the mean BR measured in (1). As to (3), we asked the subject to perform a few forced acts and then adapted the stimulus to this new BR, to allow the subjects to self-compensate for the higher tidal volume with a lower BR.

Breath and pulse (physiological signals) were recorded using a thoracic belt and a pulse oximeter, respectively, during the RT-PC acquisition, with a sampling rate of 400 Hz [31].

### 2.3. MRI Data Processing

**Figure 1:**
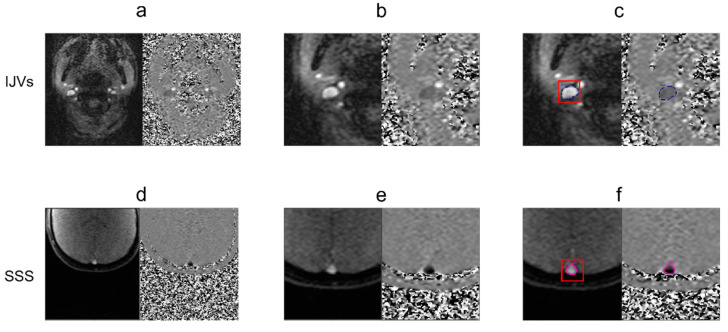
**Steps for segmenting the internal jugular veins (IJVs) (a–c) and superior sagittal sinus (SSS) (d–f).** Identification of a time point with high velocity (**a**,**d**); magnification of the vessels of interest (**b**,**e**); vessel surrounded by a segmentation box for initializing the region growing algorithm (**c**,**f**).

The RT-PC-MRI dataset was processed using the SPIN software package (SpinTech Inc., Bingham Farms, MI, USA) [32] by a single trained operator. The phase and magnitude frames containing the highest flow were selected (Figure 1a for the IJVs and Figure 1d for the SSS) and the vessels of interest were identified and magnified (Figure 1b for the IJVs and Figure 1e for the SSS). Regions of interest (ROIs) corresponding to the IJV and SSS were drawn using a semi-automated method first reported in [32]. Briefly, the semiautomatic segmentation consisted of a manual part alongside automatic processing. With the manual initialization, the user identifies the vessel of interest on the magnitude image and covers it with a box (Figure 1c,f), the center of which is around the middle of the vessel itself. That center is clicked and then used as a seed point. The software automatically segments the vessel (Figure 1c,f) using the region-growing algorithm with the full-width half-maximum threshold criterion, as explained hereafter. The mean value of the 25 pixels with the highest intensities inside the box is computed, and half of this maximum intensity is used as the threshold. Finally, the pixels where the intensity was higher than the threshold and that were connected to the seed point were included in the vessel of interest. The contours were automatically reported on the phase image that is used to confirm the segmentation correctness. The segmentation can be manually refined.

The regions of static tissue, namely, no-flow areas (NFAs), were manually drawn close to the ROIs, in order to perform background phase correction: any remnant of non-zero background phase was computed as the average phase value in the NFA. This phase offset was subtracted from the phase values of each ROI before converting the phase into velocity. The ROIs and NFA were copied over the other time frames and were manually adjusted as needed. Finally, the mean velocities inside the IJV and SSS ROIs were computed for each time frame. The IJVs and SSS cross-sectional area were then computed.

As per convention, upward-directed velocities were considered positive, while downward-directed velocities were negative. Since only venous signals were considered in this work, for an easier interpretation of the results, all the velocities were multiplied by −1. For each vessel, the average flow rate was finally computed for each time point as the average velocity over the ROI, multiplied by the vessel’s cross-sectional area.

The flow rate measure was repeated with the same method for each subject and for all the breathing scenarios.

### 2.4. Signal Analysis

The signal analysis was performed using ad hoc scripts developed in MATLAB (version 2012a, Mathworks, Natick, WA, USA). The physiologic signals were temporally aligned with the RT-PC images, using the starting time stored in the log files recorded during the acquisition and the acquisition time stored in the DICOM headers [14,31], then the average heart rate (HR) and BR during each RT-PC acquisition were computed.

For better visualization of the main flow-rate drivers, i.e., the cardiac and thoracic pumps, we plotted the breath and pulse signals, together with the flow rate, and we superimposed on the latter its band pass (0.15–0.35 Hz) filtered signal.

The temporal average flow rate was computed for each vessel and each breathing mode.

We then performed a spectral analysis consisting of the following steps: first, a fourth-grade polynomial signal detrending of the flow rates was performed; second, the power spectral density was computed. Signal detrending allowed us to avoid the high peak around zero-frequency. For each spectrum, the following peaks were observed: a high-frequency peak in the range of the BR, around 0.2 Hz; a very high-frequency peak in the range of the HR, around 1.2 Hz; and finally, another two peaks over 2 Hz corresponding to the second and third harmonics of the very high-frequency peak. For the frequency ranges, we used the conventional nomenclature employed in [33]. The first two peaks were identified, and the following frequency bands were derived: a respiratory frequency range (i.e., respiratory band) from 0.15 Hz before the high-frequency peak to 0.35 Hz after the peak, along with a cardiac frequency range (i.e., cardiac band) that was centered at the very high-frequency peak, with a range of 0.5 Hz [30,31]. The power in the respiratory and cardiac bands was computed as the area under the curve (AUC).

Then, the following indices were derived: (1) the respiratory-to-cardiac power, i.e., the AUC of the respiratory band, divided by the AUC of the cardiac band [5,30,31]; (2) the normalized respiratory (NAUC(R)) and cardiac powers (NAUC(C)), which are adimensional indexes ranging from 0 to 1, computed by dividing the AUC of the respiratory and of the cardiac bands by the whole signal variance.

### 2.5. Statistical Analysis

The statistical tests were performed using Jamovi, version 2.2 (the Jamovi project (2021), https://www.jamovi.org, accessed on 3 August 2022).

Given our sample size dimensions, descriptive statistics were expressed as median [range] values, and non-parametric tests were conducted.

Friedman tests were used in order to perform the paired statistical comparisons. The PMCMR package implemented in the Jamovi software was adopted to compute the post hoc comparisons, using the Durbin-Conover test [34] (https://cran.r-project.org/web/packages/PMCMR/index.html, accessed on 3 August 2022).

In order to assess aim 1, we tested if the following variables were significantly different across breathing scenarios (F, PN, and PD): IJVs and SSS cross-sectional area, average flow rate, and the variance of the flow rate. To confirm the correspondence between the two main peaks and the respiratory and cardiac rates, we tested the eventual frequency differences between the high-frequency peak and the BR and between the very high-frequency peak and the HR, identified separately for each breathing mode.

For aim 2, we explored whether the flow rate modulations were influenced by breathing modes (F, PN, and PD) and vessel type (IJVs and SSS). The dependent variables of this analysis were: respiratory-to-cardiac power, NAUC(R), and NAUC(C). We tested the differences among the breathing modes for each vessel, and then the differences between vessels for each breathing mode. Finally, we tested whether the NAUC(R) was significantly different compared to the NAUC(C) for each breathing mode and each vessel.

The *p*-values of all the pairwise comparisons were adjusted using a false discovery rate (FDR) correction [35]. Corrected *p*-values that were lower than 0.05 were considered statistically significant.

## 3. Results

### 3.1. Average Flow Rate and Cross-Sectional Area: Changes with Types of Breathing (Aim 1)

The image quality in the IJVs and SSS and, consequently, the estimated flow rates, was good for all the subjects.

**Figure 2:**
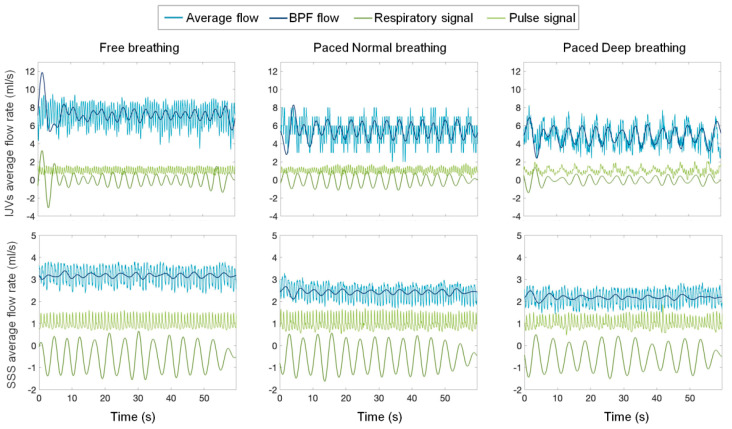
**Internal jugular veins (IJV) and superior sagittal sinus (SSS) flow rate signals of an exemplificative subject.** Different breathing scenarios, from left to right: free, paced normal, and paced deep breathing. The band pass filtered (BPF) flow-rate signal (dark blue), the respiratory signal (green), and the pulse signal (dark green) are also plotted.

An example of IJVs and SSS flow rate waveforms, superimposed onto respiratory and pulse signals, are shown separately in Figure 2 for the three breathing scenarios.

The IJVs and SSS average flow rates are reported in Table 1 as the median [range] values of the whole group of subjects, shown separately for each breathing mode.

**Figure 3:**
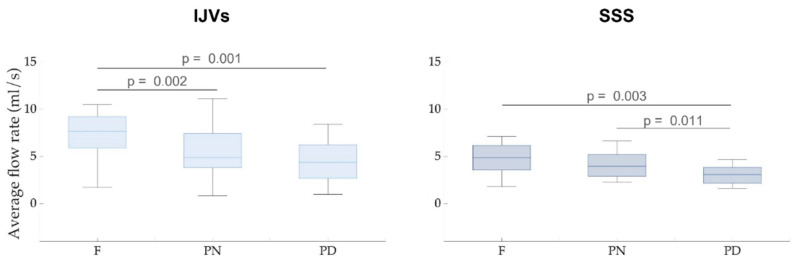
**Average flow rate boxplots for the internal jugular veins (IJVs, left panel) and the superior sagittal sinus (SSS, right panel).** The associated FDR-corrected *p*-values are written over the box plots. Legend: F = free breathing; PN = paced normal breathing; PD = paced deep breathing.

The paired FDR-corrected *p*-values are also reported. The aggregated results are also shown as boxplots in Figure 3.

For IJVs, the average flow rate was significantly decreased in the PN (p_FDR_ =0.002) and PD (p_FDR_ = 0.001) breathing scenarios, compared to the F breathing scenario. In the SSS, a significant decrease in flow rate was also found in the PD compared to the F breathing scenario (p_FDR_ = 0.003). The SSS average flow rate during the PD breathing scenario was also significantly lower, compared to the PN breathing scenario (p_FDR_ = 0.011).

No significant differences among breathing scenarios were observed, neither for the cross-sectional area nor for the flow rate variance, either for IJV or SSS (Table 1).

### 3.2. Respiratory and Cardiac Flow Rate Modulations (Aim 2)

**Figure 4:**
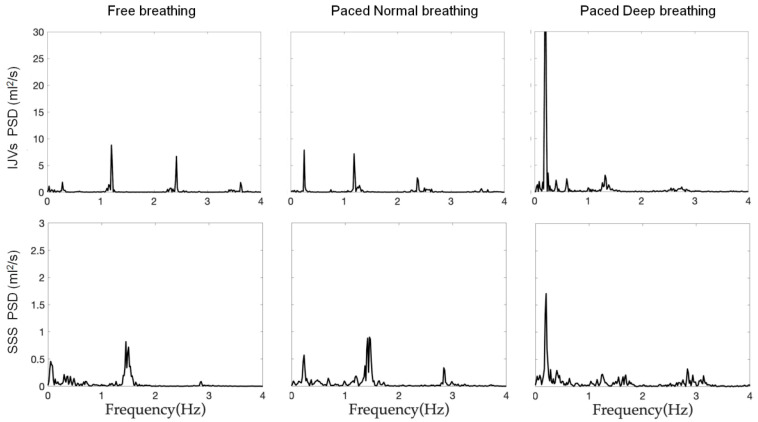
**Power spectrum densities (PSD) of one exemplificative subject’s IJVs and SSS flows.** The PSD are separately reported for each breathing scenario (from left to right: free breathing, paced normal breathing and paced deep breathing).

Exemplificative power spectral densities are reported in Figure 4, shown separately for the IJVs and SSS flow rates and for each breathing mode.

The frequencies of the two peaks that were identified in the power spectral densities were not significantly different compared to the BF (Table 2) and the HR (Table 3) for both the IJVs and SSS, for all three breathing modes.

**Figure 5:**
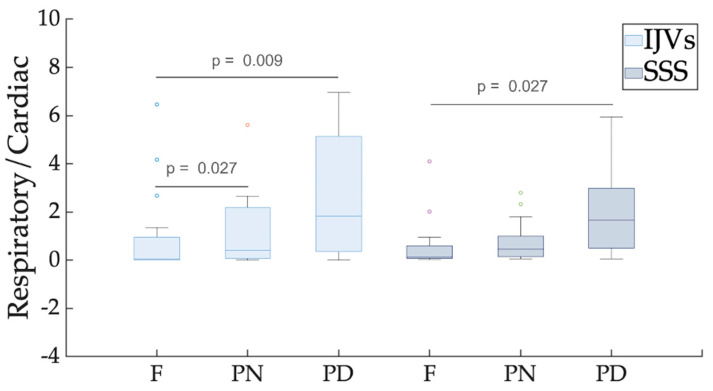
**Box plot of respiratory-to-cardiac power (Respiratory/Cardiac).** Different breathing scenarios are compared, shown separately for internal jugular veins (IJVs) and the superior sagittal sinus (SSS). FDR-corrected *p*-values are reported over the box plots, highlighting significant differences. For each breathing mode, the between-vessel comparisons were never significant.

The index of respiratory-to-cardiac power (Respiratory/Cardiac) (Table 1) significantly increased from F to PN for the IJVs (p_FDR_ = 0.027), and with a greater extent from F to PD breathing for both the extracranial, IJV (p_FDR_ = 0.009), and intracranial, SSS (p_FDR_ = 0.027), veins under consideration. Its median value was higher than 1 in the PD scenario (Table 1 and Figure 5). The comparison of this index between IJVs and SSS, recorded separately for each breathing mode, did not show any significant difference between the intra- and extracranial veins of interest.

**Figure 6:**
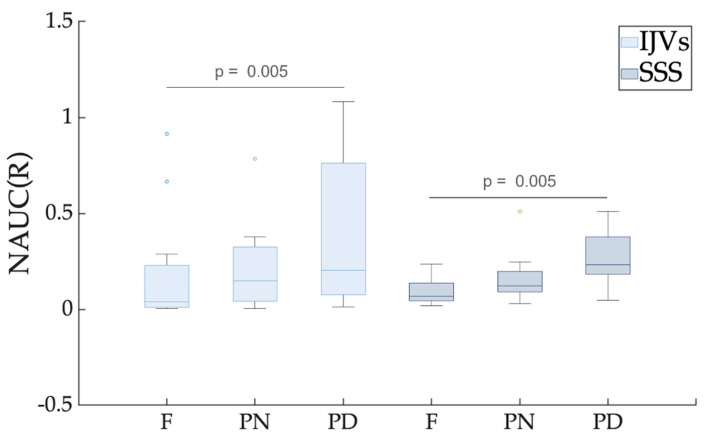
**Box plot of the normalized area under the curve (NAUC) for the respiratory component.** Different breathing scenarios are compared, shown separately for internal jugular veins (IJVs) and superior sagittal sinus (SSS). FDR-corrected *p*-values are reported for the significant paired comparisons. For each breathing mode, the between-vessel comparisons were never significant.

The NAUC(R) was significantly greater in the PD scenario compared to the F breathing scenario for both the IJVs and SSS (p_FDR_ = 0.005) (Table 1 and Figure 6).

**Figure 7:**
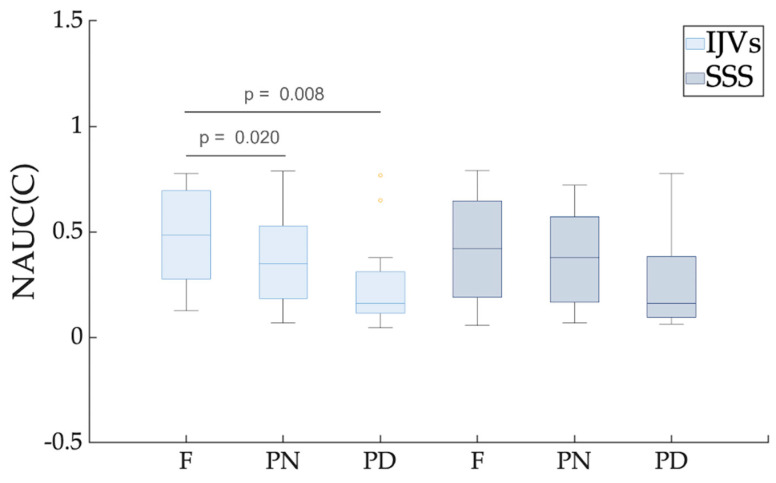
**Boxplot of the normalized area under the curve (NAUC) for the cardiac component.** Different breathing scenarios are compared, shown separately for internal jugular veins (IJVs) and superior sagittal sinus (SSS). FDR-corrected *p*-values are reported for the significant paired comparisons. For each breathing mode, the between-vessel comparisons were never significant.

The NAUC(C) was significantly smaller in the PN (p_FDR_ = 0.02) and PD (p_FDR_ = 0.008) compared to the F breathing, (Table 1 and Figure 7) for the IJVs, but no significant difference was found for the SSS.

For each breathing mode, the respiratory and cardiac contributions were not significantly different between the IJVs and the SSS.

**Figure 8:**
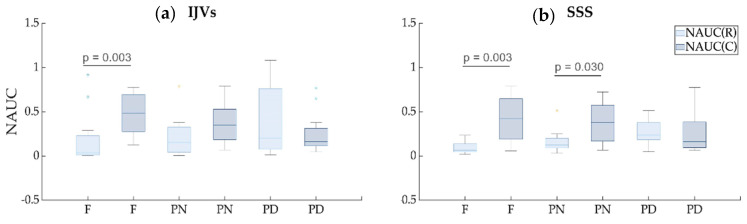
**Boxplots of the normalized AUC for the cardiac component vs. normalized AUC for the respiratory component.** The comparisons were performed for each breathing scenario (free (F), paced normal (PN), and paced deep breathing (PD)), shown separately for internal jugular veins (IJVs, panel **a**) and the superior sagittal sinus (SSS, panel **b**).

Comparing the NAUC(R) and NAUC(C) values (Table 1 and Figure 8), the latter was significantly greater compared to the former in the F breathing scenario for both the IJVs and SSS (p_FDR_ = 0.003), and in the PN breathing for the SSS scenario (p_FDR_ = 0.03). Conversely, the respiratory and cardiac contributions were not statistically different in the PN breathing mode for the IJVs and in the PD breathing for the IJVs and SSS.

## 4. Discussion

The use of RT-PC MRI to quantify the venous flow rate of the IJVs and SSS at a temporal resolution of 58.5 ms allowed us to assess how the hemodynamics were affected by the cardiac and thoracic pump. Having acquired the SSS at its median portion, it being our in-plane resolution and equal to 0.7 × 0.7 mm^2^, the SSS cross-sectional area was big enough to allow estimating good quality flow rate signal.

From a qualitative visual inspection of each 60 s acquisition (Figure 2), two main periodic oscillations were clearly visible on the flow rate curve: not only the one corresponding to the HR but also another one at a lower frequency, corresponding to the BR. The contemporaneous visualization of flow rate, breathing, and pulse signals helped to establish the breathing and cardiac modulations to the flow rate.

For quantifying these two main modulations, we conducted a spectral analysis on the flow rate, obtaining spectral density powers as those shown in Figure 4, with a high-frequency peak, a very high-frequency peak, and their second and third harmonics. The presence of the peaks over 2 Hz confirmed that the flow rate we obtained was rich in information, due to our high temporal resolution and image quality. Measuring the actual BR with the thoracic band and the HR with the pulse oximeter, we confirmed that the high-frequency peak corresponded to the BR, while the very high-frequency peak corresponded to the HR for the IJVs and SSS, as we previously showed for IJVs, internal carotid arteries, and cerebrospinal fluid flows [31]. We computed the powers in the respiratory and cardiac bands, which were normalized by the variance of the signal (NAUC(R) and NAUC(C)). The latter being approximately the area under the whole spectrum, the NAUC(R) and NAUC(C) are, therefore, adimensional indices between 0 and 1, representing the contribution of each component to flow-rate modulation.

### 4.1. Free Compared to Forced Breathing: Flow Rate Decrement

As can be observed by looking at the exemplificative flow rate across the various breathing modes shown in Figure 2, the average flow rate decreased, moving from F breathing to PN and to PD breathing, for both IJV and SSS. The statistical analyses for the whole group confirmed this observation, the average flow rate in the forced breathing being statistically lower compared to the F breathing for both the examined veins (Table 1 and Figure 3).

Different results were shown by a previous study [2] using ultrasound, where deep and free breathing were compared, but a flow rate increment was described for the IJVs only and no change was reported for the investigated intracranial veins (the Rosenthal vein and straight sinus). However, different methods and veins make direct comparison impossible for the following reasons. First, we used MRI to quantify the flow rates and the spectral analyses to quantify the respiratory and cardiac modulations, while the cited study [2] used ultrasound, with operator-dependent measures [36] in the time domain. Second, we investigated different vessels, which is another source of difference. The ultrasound study investigated the Rosenthal vein and straight sinus, which drain regions of the central brain parenchyma, rather than the SSS, which was our focus. Third, we decided to investigate the SSS, which is an intracranial vein with a larger diameter compared to those investigated in the other study because the larger a vessel is, the more reliable the flow quantification via PC-MRI [32]. Conversely, a recent study using RT-PC is in agreement with our results for the IJVs [5], as was recently also shown using the same methods in a broader group of healthy subjects [31]. As to the intracranial compartment, to the best of our knowledge, there is no previous study investigating SSS venous flow using the RT-PC MRI. However, the intracranial venous flow rate decrement in the SSS during PD breathing, compared to F breathing, mirrors the same results that were obtained for the IJVs. We hypothesize that the flow rate decrement during forced breathing may be due to higher CO_2_ blood concentrations, as shown in [37]. Indeed, we recently showed in a previous paper [31] that forced breathing may cause vasoconstriction and flow rate decrement in the internal carotid arteries, their cross-sectional areas being lower in PD breathing compared to F breathing. Consequently, the IJVs venous flow decreased, even if it occurred without a cross-sectional area change. In that previous study and also in the current one, we observed that the IJV and SSS cross-sectional areas did not change when the breathing type changed from F to PD breathing. A limitation of the present work is that we did not measure the CO_2_ partial pressure, so we cannot demonstrate that the flow rate decrement was caused by the CO_2_ partial pressure change.

### 4.2. Free Breathing Compared to Forced Breathing: Flow Rate Decrement and Respiratory Modulation Increment

We studied how the flow rate is modulated by respiration and cardiac beating during different breathing modes. In order to do that, we considered the flow-rate curve morphology, along with the whole acquisition, and extracted the oscillatory contribution of the two main drivers, i.e., respiration and cardiac beating. In particular, the powers of BR and HR oscillations were separately evaluated. Conversely, previous ultrasound studies investigating the respiratory influences on venous flow rate [2,3] considered either fiducial points (e.g., the peak velocity [2]) or average velocity and flow rates [2], or qualitatively evaluated the venous velocity modulation at the BR [3]. Our results confirmed that the extracranial venous return, via the IJVs, is highly influenced by respiration and that the respiratory modulations increased with forced breathing as previously described elsewhere [2], using ultrasound and different indices compared to ours. On the other hand, we obtained a different result from the previous study [2] for the intracranial vessels. Specifically, we established that the intracranial venous flow rate is also influenced by respiration and that the breathing modulation changed with the type of breathing, its power being a median value of 10% compared to the signal variance in the F and PN breathing, and about 20% in the PD breathing, as shown by the NAUC(R) reported in Table 1. However, we focused on a different vessel compared to those investigated by the previously cited study (i.e., SSS vs. the Rosenthal vein and straight sinus). We chose the SSS because it drains from various cortical veins [16,17], it has a crucial role in cerebrospinal fluid waste removal [18], and it has a greater diameter compared to the Rosenthal vein and the straight sinus that was investigated by Zamboni et al. [2], so we did expect to achieve a higher signal. We can find results that agree with our own, i.e., that SSS flow is modulated by respiration, looking at the figures reported in a preliminary work by Sunohara et al. [15]. Even if that study neither focused on the SSS nor commented upon the results in the area of the SSS, it did show midsagittal intracranial maps of one exemplificative subject that are useful to support our results and interpretation. The maps represented the normalized power in the respiratory band (Figure 2 of [15]) and the frequency of the highest peak (Figure 4 of [15]). Looking at the pixels in the SSS region, a high power in the respiratory band can be observed from the first map, while from the second, it is evident that respiratory frequency prevails compared to higher frequencies. This confirms that the SSS flow rate is highly modulated by respiration.

The respiratory modulation that was established for each breathing mode in our study for intra- and extra-cranial vessels increased when changing from free to forced breathing. Conversely, the cardiac modulation decreased when changing from F to PD breathing. Indeed, they are mostly complementary, comprising the main part of the flow rate variance and confirming their fundamental role as drivers. Since the variance remained stable across the various breathing modes (Table 1), a cardiac contribution decrement is expected when the respiratory contribution increases. Interestingly, the cardiac contribution prevailed in the free-breathing scenario, but it was not significantly different compared to the respiratory one in the PD scenario: in other words, the cardiac contribution decreased until it balanced the respiratory contribution in the forced breathing. For the IJVs, but not for the SSS, we observed that the two components were similar even with normal-paced breathing, meaning that a constant rate of breathing increases respiratory modulation. A greater effect of the thoracic pump was expected for the IJVs compared to the SSS, since the former represent the main drainage route of the neck, near the chest, while the latter are intracranial veins. Therefore, it is not surprising that in normal-paced breathing, the cardiac modulation prevails over the respiratory modulation for the SSS. However, using our method, we could prove that the intracranial compartment may also be influenced by respiration and by respiratory patterns. This result is not surprising, given that the SSS are connected to the IJVs through the transverse sinuses and the sigmoid sinuses. As shown in Figure 5 and in Table 1, in some subjects, the respiratory modulation prevailed over the cardiac one even in the free breathing scenario, the respiratory-to-cardiac power index being greater than one.

### 4.3. Limitations

The main limitation of the current study was its limited sample size. However, when comparing the F and the PD breathing scenarios, we obtained a significant flow rate decrement and a respiratory modulation increment in the IJVs and SSS, even after correcting for multiple comparisons. The limited sample size, as well as the imbalanced gender number, did not permit us to have better insight into cofactors such as sex, body mass index, and age. Further studies in this direction would be based on the knowledge that such factors indeed influence flow rate and the venous cross-sectional area [38,39,40,41], so that breathing and pulse modulations are also likely to be affected.

As regards the flow rate decrement, we hypothesized that this could be due to an increment of CO_2_ concentration in the blood, but in the current setup, we did not measure the CO_2_ partial pressure; therefore, we could not confirm this hypothesis. Since the same limitation was also acknowledged by another study that has just been published [5], the inclusion of CO_2_ measurement is warranted in future studies investigating blood flow changes in different breathing modes.

We also acknowledge that the blood flow changes related to the various respiratory phases (inspiration and expiration) [3] and to different breathing patterns (free or deep breathing) [2] may also be studied using ultrasound. However, the Doppler measurements obtained using ultrasound are operator-dependent, and the whole velocity trace is not usually ready to be exported in clinical settings for further analysis. The advantages of using ultrasound are that the velocity curve is immediately visualized on the screen, while the sonographer performs the examination, and the subject’s sitting position can also be explored. Although previous studies showed that neck drainage changes its pattern with different postures (supine vs. sitting) [6,7], in this study, we used an RT-PC MRI acquired on a 3T scanner; therefore we could only investigate in the supine position. Indeed, low-field scanners were used in the previously cited studies for investigating the venous vessels in different positions [6,7]. Finally, with the PC-MRI, we had to select intra- and extracranial veins that were large enough to ensure proper image quality; for this reason, we did not acquire the same vessels as those investigated using ultrasound in the previous study [2], making a direct comparison not possible.

### 4.4. Clinical Applications

Intra- and extracranial venous drainage has a crucial role in preserving brain health [29]. Quantifying the cerebrovascular circulation and its potential alterations might allow researchers to study the link between clinical alterations in aging and neurologic disorders. This is fundamental for developing new preventive and/or therapeutic strategies. Non-pharmacological therapies, such as exercise programs, have recently been described [42] for neuroprotection against cognitive decline and neurodegenerative disease as a result of aging. Another review assessed the contribution of hypoxia in neurodegenerative diseases and hypothesized that cerebral perfusion could be improved with alternate breathing or yogic interventions [43].

In the current study, we showed that respiration influences extra- and intracranial venous drainage and that venous flow rate can be altered with respect to resting conditions if the subject is asked to breathe at a constant rate at normal strength or with forced respiration.

Future studies using the setup described in the current work may contemporaneously evaluate the clinical effects of rehabilitative methods with breathing exercises and the brain’s venous drainage changes, investigating their potential link. To reduce the scanning time, the RT-PC sequence could be acquired without the initial PN breathing. Indeed, the PD works to better highlight the modulation differences, compared to the resting condition (F breathing), as can be observed from the breathing mode comparisons shown in the boxplots of Figure 5, Figure 6 and Figure 7.

## 5. Conclusions

We quantified the respiratory and cardiac modulations of venous flow rate using an innovative RT MRI technique. We showed that the breathing mode influences the average flow rate: it decreased during forced breathing, compared to normal breathing. Forced breathing also seems to influence the flow rate waveform shape, besides its average; the respiratory modulations increase and cardiac modulations decrease.

## Figures and Tables

**Table 1:** Values (median [range]) and FDR corrected *p*-values for the average flow rate, variance, area, respiratory-to-cardiac power (Respiratory/Cardiac) power, and normalized area under the curve (NAUC) for both respiratory and cardiac components, computed for internal jugular veins (IJVs) and the superior sagittal sinus (SSS).

Breathing Modes			pFDR			
	F	PN	PD	F vs. PN	F vs. PD	PN vs. PD
**Average flow rate IJVs (mL/s)**	7.66 [1.72–10.50]	4.88 [0.82–11.10]	4.39 [0.99–8.41]	**0.002**	**0.001**	0.132
**Average flow rate SSS (mL/s)**	4.87 [1.84–7.13]	3.97 [2.30–6.65]	3.08 [1.64–4.69]	0.380	**0.003**	**0.011**
**Variance flow rate IJVs (mL/s)^2^**	1.97 [0.46–2.88]	1.19 [2.73–9.68]	1.50 [0.02–10.40]	0.090	0.188	0.732
**Variance flow rate SSS (mL/s)^2^**	0.21 [0.02–0.92]	0.15 [0.02–0.59]	0.16 [0.03–0.49]	0.430	0.890	0.470
**Area IJVs (mm^2^)**	28.70 [21.50–40.70]	22.20 [7.94–71.50]	24.50 [5.14–69.20]	0.914	0.914	0.913
**Area SSS (mm^2^)**	21.50 [14.50–43.50]	27.30 [13.10–40.20]	24.10 [14–47.20]	0.913	0.963	0.914
**Respiratory/Cardiac IJVs**	0.056 [0.004–6.460]	0.415 [0.004–26.900]	1.830 [0.015–18.200]	**0.027**	**0.009**	0.128
**Respiratory/cardiac SSS**	0.135 [0.029–4.090]	0.451 [0.045–2.800]	1.660 [0.062–5.950]	0.430	**0.027**	0.128
**NAUC(R) IJVs**	0.27 [0.06–1.62] a	0.32 [0.15–0.69]	0.53 [0.27–0.79]	0.070	**0.005**	0.175
**NAUC(R) SSS**	0.12 [0.04–0.38] b	0.13 [0.04–0.31] c	0.19 [0.07–0.32]	0.138	**0.005**	0.140
**NAUC(C) IJVs**	0.48 [0.13–0.77] a	0.35 [0.07–0.79]	0.16 [0.05–0.77]	**0.020**	**0.008**	0.150
**NAUC(C) SSS**	0.42 [0.06–0.79] b	0.38 [0.06–0.72] c	0.16 [0.06–0.78]	0.520	0.087	0.250

F = free breathing; PN = paced breathing; PD = paced deep breathing. NAUC(R) vs. NAUC(C) comparison: (a) and (b) *p* = 0.003; (c) *p* = 0.03.

**Table 2:** Comparison of high-frequency peak and respiratory frequency: the median [range] values are reported for each breathing mode and vein, along with the FDR-corrected *p*-value of the statistical comparison.

	Breathing Mode	High-Frequency Peak	Respiratory Frequency	Respiratory Frequency vs. High-Frequency Peak pFDR
**IJV**	**F**	0.24 [0.20–0.35]	0.26 [0.22–0.31]	0.126
	**PN**	0.25 [0.20–0.40]	0.25 [0.20–0.40]	0.790
	**PD**	0.21 [0.16–0.35]	0.22 [0.18–0.24]	0.790
**SSS**	**F**	0.26 [0.15–0.35]	0.25 [0.19–0.32]	0.493
	**PN**	0.25 [0.20–0.28]	0.25 [0.20–0.26]	0.288
	**PD**	0.22 [0.17–0.25]	0.22 [0.17–0.25]	0.288

**Table 3:** Comparison of very high-frequency peak and cardiac frequency: the median [range] values are reported for each breathing mode and vein, along with the FDR-corrected *p*-value from the statistical comparison.

**Vein**	**Breathing Mode**	**Very High-Frequency Peak (Hz)**	**Cardiac Frequency (Hz)**	**Cardiac** **Frequency vs. Very High-Frequency Peak p_FDR_**
**IJV**	**F**	1.29 [0.80–2.82]	1.22 [0.80–1.69]	0.880
	**PN**	1.27 [0.82–2.72]	1.29 [0.81–1.65]	0.900
	**PD**	1.21 [1.03–1.80]	1.39 [0.83–1.90]	0.889
**SSS**	**F**	1.29 [0.79–1.74]	1.32 [0.77–1.74]	0.199
	**PN**	1.24 [0.82–1.62]	1.34 [0.85–1.64]	0.190
	**PD**	1.39 [0.59–1.74]	1.38 [0.55–1.52]	0.190

## Data Availability

The data presented in this study are available on request from the corresponding author.
